# Exploring non-medical prescribing for patients with mental illness: a scoping review

**DOI:** 10.1186/s12888-025-06938-6

**Published:** 2025-05-19

**Authors:** Bashayr A Alsaeed, Jason Hall, Richard N. Keers

**Affiliations:** 1 Division of Pharmacy and Optometry, School of Health Sciences, Faculty of Biology, Medicine and Health, Manchester Academic Health Science Centre, The University of Manchester, Manchester, UK; 2 NIHR Greater Manchester Patient Safety Research Collaboration, University of Manchester, Manchester, UK; 3 Optimising Outcomes With Medicines (OptiMed) Research Unit, Pennine Care NHS Foundation Trust, Greater Manchester, UK; 4 Department of Clinical Pharmacy, College of Pharmacy, Jouf University, Al-Jouf Sakaka, 72341 Saudi Arabia

**Keywords:** Non-medical prescribing, Nurse prescriber, Pharmacist prescribers, Mental health

## Abstract

**Background:**

Non-medical prescribing (NMP) qualifications extend prescribing authority to nurses, pharmacists, and other allied health professionals and are advocated as a means to help improve healthcare efficiency and access to services. However, despite non-medical prescribers (NMPs) being utilised in some countries for more than two decades, less is known about their role and impact in the mental health context. This scoping review therefore aims to map published research evidence concerning NMP for patients with mental illness.

**Methods:**

Five electronic databases were searched from January 2003 to July 2024. Reference lists of identified papers were also checked for relevant studies. Full text primary research studies exploring the nature, impact, and implementation of NMP services for patients with mental illness and dementia in community settings were included.

**Results:**

From 22,547 retrieved papers, 63 studies were included. Of these, 57 (90.4%) detailed the nature (including the service or team they were based in, medicines prescribed, and main role(s)) of NMPs, 45 (71.4%) assessed the impact of services, and 16 (25.3%) explored factors influencing service implementation and delivery. The majority of studies originated from the USA (30/63, 47.6%), or the United Kingdom (27/63, 42.8%). Either nurse (44/63, 69.8%), pharmacist (16/63, 25.3%) or non-medical prescribing models featuring both professionals were exclusively studied (3/63, 4.7%). In the UK and USA, antidepressants (60%, 54.5%) were the most prescribed by NMPs. Although the conditions of patients managed by both nurse and pharmacist prescribers were reported to be well managed based on clinical outcomes (3/24, 12.5% and 3/13, 23%, respectively), few studies evaluated health outcomes. Training-related barriers to service delivery were commonly mentioned in UK studies (4/9, 44.4%), with financial issues reported in the USA (2/4, 50%).

**Conclusions:**

This review highlights the diverse roles of NMPs in the care of people with mental illness. Whilst the limited evidence suggests a positive impact on patient care, more quantitative research is needed. Given the focus on nursing prescriber models in this review, along with rising mental health demand and continuing staff shortages, future research should prioritise exploring and evaluating the contribution of pharmacist NMP services as well other healthcare professionals' NMPs.

**Supplementary Information:**

The online version contains supplementary material available at 10.1186/s12888-025-06938-6.

## Background

There is currently high demand for mental health services across all age groups globally [1]. This trend was exacerbated by the COVID-19 pandemic, which saw increased numbers of children and adolescents in particular seeking help for mental health issues [2]. This surge in demand has also coincided with a shortage of staff to provide the necessary services [3]. The World Health Organization has acknowledged that high demand for services has led to difficulties in many countries adequately meeting this growing need [4]. Tackling workforce challenges has thus been identified as one of the main priorities in terms of enhancing the care provided to patients, based on improving access to and timely provision of safe services [5].

Non-medical prescribing (NMP) involves the use of trained healthcare professionals other than doctors, such as pharmacists and nurses, to prescribe medications; it has emerged as an approach to help alleviate workforce challenges, to expand access to care, and to address high demand [6]. The main drivers for the introduction of NMP services are the need to streamline access to medications and related services, promote full utilisation of healthcare professionals' skills, and facilitate interprofessional collaboration within the healthcare service to provide more comprehensive, high-quality care to patients [6]. NMP takes the form of two main models; supplementary prescribing (supervised prescribing), where the prescriber may collaborate with a physician to manage patient care [7], and independent prescribing, in which the non-medical prescriber (NMPs) may assess patient's condition and prescribe medicines autonomously. Independent prescribing is distinguished from supplementary prescribing, with training in diagnoses being one of the main differences [8]. Other differences that distinguish independent from supplementary prescribing include greater autonomy and the ability to prescribe without a pre-agreed clinical management plan [8]. The degree of autonomy in supervised prescribing models (such as supplementary prescribing in the UK) varies across countries. This includes collaborative agreements, restricted, and reduced practice models, which limit prescribing authority based on supervision, medication types, or clinical settings [9, 10]. The non-medical professions that are permitted to prescribe vary across countries and are usually authorised following completion of specific training. For example, in the UK, nurses complete a Nursing and Midwifery Council Independent Nurse Prescribing Course (V200/V300)) [11]. Whereas in most of USA states as well as in other countries (e.g., Australia, Canada, Sweden, Finland, Ireland, the Netherlands, New Zealand, and Spain) non-medical prescribing is also allowed, albeit with varying restrictions [12]. Pharmacists in the UK who have completed further training can also prescribe autonomously, whilst in the USA, pharmacists can prescribe with restrictions in some states; pharmacist prescribing roles are still under review in some other countries [12]. Although NMP has been implemented in different forms globally, the UK's prescriptive authority for NMPs is among the most comprehensive [13]. UK non-medical prescribers can prescribe for all conditions and medicines, including controlled substances, while other countries limit prescribers to specific formularies and conditions [13–15].

Since the first initiation of NMP services in the USA in the 1960s [13], several evaluation studies focusing on patients with widely observed conditions such as diabetes, skin conditions, acute respiratory tract infections, and hypertension, have been carried out across various care settings, including general practice (also known as family medicine or primary care in some countries) and community pharmacies [16–19]. Patients with diabetes managed by nurse prescribers have highlighted the comprehensive care provided and noted their enhanced access to medications [16]. Patients treated for skin conditions by pharmacist prescribers similarly reported positive benefits [17]. According to one review article published in the UK, the positive impact of NMP services goes beyond improving patient care; it may also positively affect other healthcare professionals, enabling medical prescribers to use their time more effectively so they can focus on complex cases [12]. Despite evidence of positive outcomes from NMP services, several practical barriers to both implementation and delivery have been encountered. A systematic review of 42 studies, published in 2018, reported a perceived lack of support among NMPs after completion of their training which involved a lack of clinical supervision and mentoring. Additionally, the review reported limited standardised national policies and guidelines in place for NMPs to follow which may have hindered optimal implementation within general practice [20].

In the context of mental health care, positive outcomes have also been reported by patients with mental illness in receipt of NMP services. From a patient perspective, an interview study in the UK from 2011 reported that getting access to mental health nurse prescribers was seen to be easier and quicker compared to medical prescribers [21]. A qualitative systematic review of 12 studies, published in 2017 which explored nurse prescribing in mental health also observed that the use of mental health nurse prescribers enabled patients to be more involved in decision-making than the standard care provided by a medical prescriber [22]. However, while medical prescribers’ views on mental health nurse prescribing were largely positive in a UK study from 2015 [23], in another UK-study, some psychiatrists, reported concerns that while nurses were capable of prescribing some medications, they should not be granted full prescribing authority [24]. Evidence from NMPs in mental health care also suggests that limited support, unclear specification of their roles, and being underpaid as compared to doctors may be barriers to them taking on prescribing roles [25].

Despite the fact that there have been some published studies concerning the work of NMPs in relation to patients with mental illnesses [21, 23–26], the literature is disparate, and there have been few attempts to integrate such studies in order to develop more informed recommendations for clinical practice and future research. One qualitative systematic review, published in 2017, aimed to identify and summarise qualitative evidence focusing solely on mental health nurse prescribing; this requires updating and also did not consider evidence regarding the impact of wider NMP services (e.g., on healthcare services and other professionals) [22]. Additionally, the available evidence from relevant reviews highlights a lack of understanding of NMP services delivered by other professionals, such as pharmacists. Conducting an up-to-date and comprehensive review is therefore necessary to enable full understanding of the contextual reality of NMP services for people with mental illness.

The aim of this scoping review was to map the existing global published evidence on NMP services for patients with mental illness with a focus on the nature of such services, their impact, and the factors influencing their successful implementation and delivery. It is hoped that the findings of this review will inform the development of recommendations to both support the future optimisation of services for patients with mental illness and to help identify research priorities.

## Method

The method used in this scoping review was based on the framework initially established by Arksey and O'Malley, as further enhanced by Levac, Colquhoun, and O’Brien [27, 28].

### Identifying the research question

The three research questions identified:

1. What is the role(s) of non-medical prescribing with regard to patients with mental illnesses?
2. What is the impact of non-medical prescribing on patients with mental illnesses?
3. What are the factors influencing the successful implementation and delivery of non-medical prescribing services for patients with mental illnesses?

### Identifying relevant studies

#### Data source

Five electronic databases were searched: Embase, Medline, PsycINFO, CINAHL PLUS, and Web of Science. These are key databases known to provide comprehensive coverage of the medical and allied health care professional literature [29, 30]. The reference list of all included papers was also checked manually. Only primary literature was included, encompassing qualitative, quantitative, and mixed-methods studies. The grey literature was not searched and no hand searching of individual journals was carried out. The reason for excluding the grey literature was the priority of including high-quality research, which the grey literature might not provide.

#### Search strategy

Database search terms were categorised into four themes: “non-medical”, “mental health”, “prescribing”, and “evaluation”. Several variations of words related to the root themes were then used. Boolean operators (AND, OR) and truncation (where appropriate) were used. The search terms were discussed with a specialist librarian to develop a comprehensive search strategy. The same search terms were utilised across each database, with minor amendments only due to the different database syntaxes. The search strategy used for one database can be found in *Supplementary 1*. No restrictions on language and subject were implemented while searching. The search was conducted from 1 January 2003 to 8 July 2024. The initial search date of 2003 was chosen to reflect the amendment in UK legislation in 2003 that first extended prescriptive authority, enabling nurses and pharmacists to prescribe as supplementary prescribers for several medical conditions, including mental health issues [31]. A Preferred Reporting Items for Systematic Reviews and Meta-Analyses Extension for Scoping Reviews (PRISMA-ScR) flow diagram was used to document and summarise the screening process including reporting the number of papers used at different stages of the screening process; this was also used to report the findings [32]. The (PRISMA-ScR) checklist can be found in *Supplementary *2.

#### Study selection

**Fig. 1:**
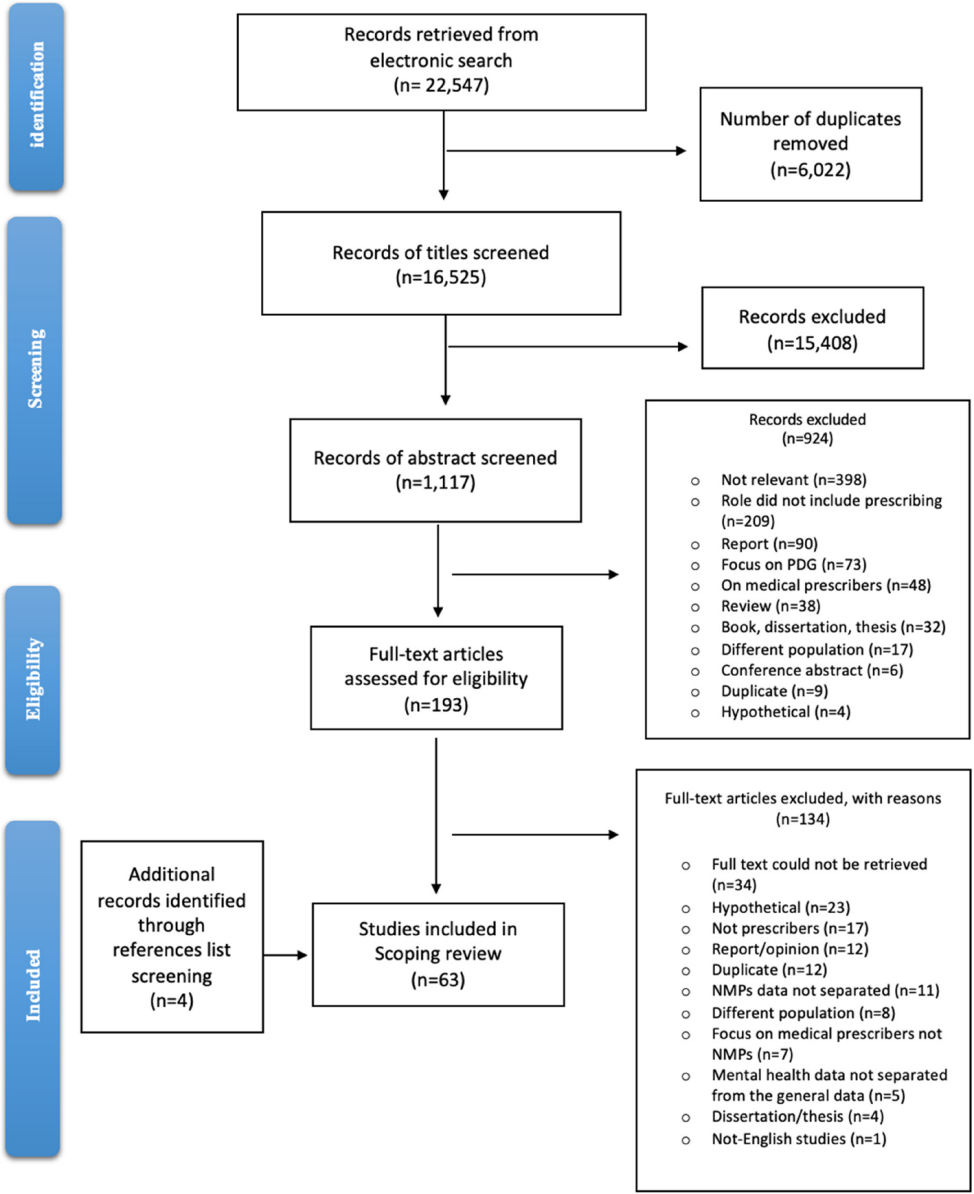
PRISMA-ScR) flow diagram of identification, screening, and selection process

After exporting all the identified studies from electronic databases to the software manager (EndNote 20) and removing any duplicates (EndNote was used to identify duplicates, followed by a manual review to ensure accuracy), three levels of sequential screening were used to assess the relevance of the studies against the eligibility criteria (Table 1). One reviewer (B.A.) independently screened the titles then abstracts. Any uncertainty about any study was discussed with the wider research team regarding inclusion or exclusion. Following this, remaining papers were subject to full-text screening. The reasons for excluding studies at the abstract and full-text stages are explained in the PRISMA-ScR flowchart (Fig. 1).

**Table 1:** Inclusion and exclusion criteria

Inclusion criteria	Exclusion criteria
Primary research studies (including qualitative, quantitative, and mixed-methods research) that reported on at least one of the following: the nature and/or evaluated the impact and/or identified the barriers & facilitators of NMP service for patients with mental illnesses	Studies focused on patient group directions, as they involved only the supply/administration of medicines without requiring a prescription
Primary research studies published from 2003–2024	Case study reports/ literature review studies/ conference abstracts
Primary research studies describing non-medical prescribing for patients with mental illnesses regardless of the health care setting	Books, dissertation, and thesis documents
Primary research studies focused on either one or more broad medication classes	Studies focused solely on substance use and addiction disorders
Primary research studies focused on a single drug. This to understand more broadly NMP services for patients with mental illness rather than solely focusing on the specific medications used	Studies that focused on the attitudes and beliefs toward a hypothetical service rather than being based on the actual delivery of NMP services
Primary research studies on dementia were included. This is because patients with dementia may commonly receive care within mental health services [33]	Studies where data relating to NMPs could not be separated from other medical prescribers
Patients with substance use disorder/addiction as dual diagnosis with mental illness	Non-English language studies

#### Charting the data

A data charting form was developed by one reviewer (B.A.) as shown in *Supplementary file 3*. The key variables collected from each study were as follows: study title, name of the author(s), year of publication, study country, study population (number of non-medical prescribers, profession of non-medical prescriber), study setting, study method, and the aim of the study. Where present, information regarding the nature of NMPs, such as what medication they were prescribing, what service(s) they provided, and what mental health illnesses they were dealing with, was gathered, along with the impact of NMP services on, for example, patient care, medical prescriber(s)/other healthcare professionals, and wider services. Finally, any factors influencing the implementation and delivery of NMP services were identified. In order to ensure the validity of the extraction process, after data was extracted by the first reviewer (B.A.), data from a total of 20 out of 63 included papers were independently double-extracted by the wider research team (RN. K, J.H).

#### Collating, summarising, and reporting the results

The key information from each included study was summarised in a table of characteristics (Table 2). These characteristics were author, year, country, setting (community care or hospital), study design and methods, subjects (nurses, pharmacists, or both), and whether the study reported information on the role of NMPs, the impact of NMP, and/or factors influencing successful implementation and delivery of NMP services for patients with mental illnesses. The key findings from each of these three objectives were presented separately both in tabular form and descriptively. Due to the differences in healthcare systems between countries, the settings were classified as “community care” and hospital care”. Community care referred to any care delivery setting where patients were treated without being hospitalised, such as general practices, outpatient facilities, and community health centres. Whereas hospital care involved inpatients receiving services from NMPs. Some studies discussed prescribing in the traditional sense, such as writing new prescriptions. Other studies focused on what they described as ‘management’, where prescribing occurred as part of an agreed management plan. For consistency, we used the term "prescribing" to refer to both scenarios collectively.

**Table 2:** A summary of included study characteristic

Study Author/Year	Country of origin	Study setting	Study design/ Methods	Subject	Participant number	Role of NMP described?	Impact of non-medical prescribing services described?	Barriers of non-medical prescribing services described?	Facilitators to non-medical prescribing services described?
Bo Kyum et al. (2017) [34]	USA	CC	Quantitative (The National Ambulatory Medical Care Survey)	NP	920 patients on NG and 5,373 patients on MG	√	Χ	Χ	Χ
Bradley et al. (2008) [35]	UK	Single mental health trust	Qualitative (Focus group)	NP	15 (8 nurse prescribers /7 non-prescriber)	Χ	√	Χ	Χ
Brimblecombe et al. (2022) [36]	UK	Both	Quantitative (Time series questionnaire)	NP		√	Χ	√	√
Brodie et al. (2014) [37]	UK	CC	Qualitative (Face to Face interviews)	NP/PP	4 NP, 4 PP	√	Χ	√	Χ
Buist et al. (2019) [38]	UK	CC	Mixed (Questionnaire with patients / interviews with pharmacists and MDT)	PP	75 patients	√	√	√	Χ
Cai et al. (2022) [39]	USA	CC	Quantitative (National Medicare claims database)	NP	608,787 patients on NG and 1,290,026 patients on MG	√	Χ	Χ	Χ
Chung et al. (2011) [40]	USA	CC	Quantitative (Patients records, and survey)	PP	34 patients	√	√	Χ	Χ
Delaney et al. (2019) [41]	USA	Both	Quantitative (Survey)	NP	1,624 PMH APRN	√	Χ	Χ	Χ
Deslandes et al. (2015) [42]	UK	CC	Qualitative (Semi-structured interviews, self-completion diaries)	PP	11 patients	√	√	Χ	Χ
Dobel-Ober et al. (2013) [43]	UK	Both	Qualitative (Semi-structured interviews)	NP	20 NP	√	√	Χ	√
Earle et al. (2011) [44]	UK	CC	Qualitative (Semi-structured interviews)	NP	3 nurses, 1 each of psychiatrist, occupational therapist, nutritionist, psychologist, social worker, and support worker	Χ	√	Χ	Χ
Earle et al. (2011) [21]	UK	CC	Qualitative (Semi-structured interviews)	NP	2 CPN and 6 patients	√	√	√	Χ
Feldman et al. (2003) [45]	USA	CC	Mixed (Survey, psychotropic drug claims, clinical record review)	NP	For the survey: 2,335 ANCC For psychotropic drug claims: 787 (APPNs in 15 states) + 247 (APPNs associated with clinic practices) + 2,295 (psychiatrists) = 3,329 participants in total	√	Χ	Χ	Χ
Foreman et al. (2011) [46]	UK	CC	Quantitative (Questionnaires)	NP	36 patients on NG, 34 on MG	√	√	Χ	Χ
Frain et al. (2021) [47]	The Republic of Ireland	CC	Qualitative (Semi-structured interview)	NP	12 patients	Χ	√	Χ	Χ
Graham et al. (2020) [48]	UK	CC	Qualitative (Individual, semi-structured interview)	PP	4 nurses, 1 psychologist and 1 psychiatrist, and 3 people with caring responsibilities for persons with learning disabilities	√	√	Χ	Χ
Green et al. (2008) [49]	UK	Both	Quantitative (Mail survey)	NP/PP	12 respondents	√	√	Χ	√
Gumber et al. (2011) [50]	UK	CC	Quantitative (Questionnaire)	NP/PP	8 Nurses, and 2 pharmacists	√	√	Χ	Χ
Harvey et al. (2018) [51]	Australia	CC	Quantitative prospective (Using (EDS), (PCS), and (HONOS) scale)	NP	221 Patients	√	√	Χ	Χ
Jones et al. (2007) [52]	UK	NA	Qualitative (Semi-structured interview)	NP	12 psychiatrists, 11 MHNP, and 12 patients	√	√	√	Χ
Kaye et al. (2009) [53]	USA	CC	Mixed (Survey)	NP	58 county representatives	√	√		Χ
Klein et al. (2016) [54]	USA	CC	Quantitative (Pharmacy claims dataset)	NP	10,753 children included who received the 82,754 complete 30-day prescriptions from 1785 unique prescribers (78 NP specialists; 303 NP generalists; 162 physician specialists; and 1242 physician generalist	√	Χ	Χ	Χ
Kozlov et al. (2018) [55]	USA	HC	Quantitative (survey)	NP	236 IPCP	√	Χ	Χ	Χ
Martinson et al. (2010) [56]	USA	CC	Quantitative (A survey)	NP	42 FNPs	√	Χ	Χ	Χ
Nolan et al. (2004) [57]	USA	Both	Mixed (A survey)	NP	51 nurses	√	√	Χ	Χ
Norman et al. (2010) [58]	UK	Both	Quantitative (post-test control group experimental) and qualitative (MARS, SIMS, WSAS, The Beck Depression Inventory-II, CGI, CSQ-8, Structured interview)	NP	45 patients for NG and 45 patients for MG	√	√	Χ	Χ
Oldknow et al. (2020) [59]	UK	One NHS trust	Qualitative (mapping exercise, Trust policy documents analysis, interview)	NP	6 MHN	Χ	√	√	Χ
Page et al. (2008) [60]	UK	CC	Qualitative (Interview)	NP	7 patients of the memory clinic, 6 family carers and 7 staff members	√	√	Χ	Χ
Park et al. (2022) [61]	USA	CC	Quantitative (IBM MarketScan commercial and Medicare supplemental claims)	NP	A total of 103,067 AD prescriptions	√	Χ	Χ	Χ
Ross (2015) [23]	UK	CC	Qualitative (questionnaire for demographic data, focus group, interview)	NP	35 NP, 3 PP, 2 NM, 7 CP, 1 GP and 9 clients	Χ	√	Χ	Χ
Ross et al. (2012) [25]	UK	Both	Mixed (questionnaire, focus group)	NP	33 MHN for the questionnaire, and 12 NP for the focus group	√	√	√	Χ
Chetan et al. (2021) [62]	UK	CC	Mixed (Self-administrated survey, focus group)	PP	For the survey: 20 from the carer for CAYP with ADHD, 13 MDT (4 psychiatrists, 3 psychologists, 3 administrative staff, 2 nurses and 1 social worker) NA for the focus group	√	√	Χ	Χ
Silvia et al. (2020) [63]	USA	CC	Quantitative (Retrospective chart review, patient survey)	PP	107 patients in PP and 34 patients in BHCP	√	√	Χ	Χ
Snowden A (2013) [64]	UK	NHS Greater Glasgow and Clyde	Qualitative (Semi-structured interview)	NP	11 RMNs	Χ	√	√	Χ
Snowden A (2008) [65]	UK	NHS Greater Glasgow and Clyde	Quantitative (Questionnaire)	NP	11 RMNs	√	√	Χ	Χ
Snowden A (2010) [66]	UK	Both	Mixed (survey)	NP	42 MHNPs	√	√	Χ	Χ
Louise S (2010) [67]	UK	CC	Quantitative (survey)	NP	49 Patients	√	√	Χ	Χ
Williams et al. (2009) [68]	USA	CC	Mixed (Survey, interview)	NP	56 patients and 13 MP and 10 NP	√	√	Χ	Χ
Stuart W (2007) [69]	UK	CC	Mixed (survey)	NP	78 patients for the survey 2 supervising consultant psychiatrists	√	√	Χ	Χ
Wolfe et al. (2008) [70]	USA	Both	Quantitative (questionnaire)	NP	183 PN	√	Χ	Χ	Χ
Yang et al. (2018) [71]	USA	CC	Quantitative (Annual computerized administrative claims data)	NP	1,034,798 psychotropic medications dispensing for 61,526 individual youths (89,787 treated by MP, 8118 by NP, and 15,935 by both)	√	Χ	Χ	Χ
Yang et al. (2021) [72]	USA	CC	Quantitative (Medicaid -insurance claims data)	NP	12,991 individuals included in this study (Individuals treated by PSs (n = 6317), in which 5832 treated by Psychiatrists, and 485 by Psychiatric NPs, whereas (n = 6674) treated by PCPs, in which 5819 treated by physicians, and 855 treated by NPs	√	Χ	Χ	Χ
Adler et al. (2019) [73]	US	CC	Quantitative (survey)	NP	702 HCPs (201 psychiatrists, 201 PCPs, 200 neurologists, and 100 NPs)	√	Χ	Χ	Χ
Araya et al. (2009) [74]	Ethiopia	HC	Quantitative (questionnaire)	NP	32 nurses and 55 colleagues	√	√	√	Χ
Mitchell et al. (2016) [75]	Australia	CC	Mixed (Single multidisciplinary case conference, semi-structured interview, In-depth interviews, telephone interviews series of validated questionnaires, full cohort evaluation)	NP	6 patients/careers for the questionnaire and semi-structured interview NA for In-depth interviews 2 telephone interviews with SC 23 case conferences	√	√	Χ	Χ
Oldknow et al. (2010) [76]	UK	CC	Mixed (Face to face interview, survey, document analysis)	NP	3 SN, and 4 CPs for the interview. 16 patients for the questionnaires	√	√	Χ	Χ
Chapman et al. (2019) [9]	US	Both	Qualitative (Site interview, review of state scope of practice regulation)	NP	94 interviews with state BON staff, APN org. staff, PMHNP edu. program directors, program faculty, PMH-APRNs, Ag/Sys directors, psychiatrists, and OHPs who worked with PMH-APRNs	√	Χ	√	Χ
Phoenix et al. (2016) [77]	US	CC	Qualitative (Semi-structured interview)	NP	50 with CMHD, MHMD, PMHNPs, HRM, QM and FBM	√	√	√	Χ
Wortans et al. (2006) [78]	Australia	CC	Qualitative (Semi-structured interview)	NP	7 patients	√	√	Χ	Χ
Xu et al. (2020) [79]	Singapore	CC	Quantitative (Questionnaire, retrospective review of medical records)	NP	100 patients	√	√	Χ	Χ
Baum et al. (2023) [10]	USA	Both	Mixed (Survey, DEA-Registered VA Pharmacist Data Analysis, Time and Motion Analysis)	PP	87 DEA-Registered VA Pharmacists	√	Χ	Χ	Χ
Gallagher et al. (2023) [80]	USA	CC	Quantitative (survey)	NP	400 Nurse practitioners	√	Χ	√	√
Gibu et al. (2017) [81]	USA	CC	Quantitative (Database and the Computerized Patient Record System, the VA’s electronic medical record)	PP	81 patients	√	√	Χ	Χ
Goodman et al. (2023) [82]	USA	CC	Mixed (report analysis, surveys)	PP	3 Mental Health clinical pharmacist practitioners	√	√	Χ	Χ
Hammersla et al. (2024) [83]	USA	CC	Quantitative (Medical Record Review, medication Reconciliation, the Anticholinergic Burden Score)	PP	88 patients	√	√	Χ	Χ
Kallis et al. (2023) [84]	UK	CC	Qualitative (semi-structured interview)	PP	6 Clinical pharmacists	√	Χ	√	Χ
Kosar et al. (2024) [85]	USA	CC	Quantitative (Medicare claims data, surveys, Area Health Resources File)	NP	334 618 Nursing home decedents with ADRD	√	√	Χ	Χ
Maryan et al. (2019) [86]	USA	CC	Mixed (prospective chart review, survey, Glasgow Antipsychotic Side-effects Scale for Clozapine (GASS-C))	PP	Patients (11 patients were seen in PP and MP group)	√	√	√	√
May et al. (2023) [87]	UK	CC	Mixed (Questionnaires)	NP	23 Patients	√	√	√	Χ
Nulph et al. (2023) [88]	USA	HC	Quantitative (medical records)	PP	NA	√	√	Χ	Χ
Olson et al. (2023) [89]	USA	CC	Quantitative (medical records)	PP	Patients (111 on pharmacist group, and 110 on Psychiatrist group)	√	√	Χ	Χ
Chavez et al. (2019) [90]	USA	CC	Quantitative (report analysis, survey)	PP	2 2 bilingual clinical pharmacists, 47 patients	√	√	Χ	Χ
Stuart et (2020) [91]	USA	HC	Quantitative (A standardized case report form)	PP	79 Patients	√	√	Χ	Χ

*NP* Nurse prescriber, *PP* Pharmacist prescriber, *NA* Not available, *CC* Community Care, *HC* Hospital Care, *NG* Nurse Group, *MG* Medical Group, *CPN* Community Psychiatric Nurses, *AD* Alzheimer Disease, *PSs* Psychiatric Specialists, *PCPs* Primary care providers, *CMHD* County Mental Health Director, *MHMD* Mental health medical director, *PMHNPs* Psychiatric Mental Health Prescribers, *HRM* Human Resources Manager, *QM* Quality manager, *FBM* Financial and billing manager, *APPNs* Advanced Practice Psychiatric Nurses, *MARS* The Medication Adherence Report Scale, *SIMS* The Satisfaction with Information about Medicines Scale, *WSAS* The Work and Social Adjustment Scale, *CGI* The Clinical Global Impression of Improvement scale, *CSQ-8* The eight item Client Satisfaction Questionnaire, *CAYP* Child and Young People, *SC* Senior Clinicians

Due to variations across countries, the models of prescribing were also classified into two main categories: “independent prescribing” and “supervised prescribing”. Supervised prescribing was used to refer to any form of supervision by a designated medical professional required, this including supplementary prescribing in the UK and restrictive/reduced practice, and collaborative practice agreements in the USA and other countries. Independent prescribing, in contrast, referenced NMPs’ ability to prescribe medication autonomously without any form of supervision. The various models of supervised practice are described in *Supplementary file 4.*

## Results

In total, 22,547 papers were retrieved from the five electronic databases searched. A total of 6,022 duplicates were then identified through EndNote and removed, leaving 16,525 articles to be screened by title. After title screening, 1,117 articles remained for abstract screening, During full-text screening, 134 further papers were excluded, primarily because the full text could not be retrieved, the study focused on hypothetical services, or the role/service described did not involve non-medical prescribers. Four additional studies were identified by hand screening the reference lists of the included papers (59), which resulted in 63 papers being included in the final review. Details for the three stages of the screening process and reasons for exclusion at the full-text review stages are demonstrated in a PRISMA-ScR flow diagram chart (Fig. 1).

### Characteristics of included studies

Of the 63 included studies, 57 (90.4%) reported on the nature of NMP services for patients with mental illness [9, 10, 21, 25, 34, 36–43, 45, 46, 48–58, 60, 62, 63, 65–92], 45/63 (71.4%) reported on the impact of NMP services delivered to patients with mental illness [21, 23, 25, 35, 38, 40, 42–44, 46–53, 57–60, 62–69, 74–79, 81–83, 85–91], and 16 (25%) reported on factors influencing successful implementation and delivery of these services. Of those 16 studies, 14 (22.2% of all studies examined) reported barriers to implementation and delivery [9, 21, 25, 36–38, 52, 59, 64, 74, 80, 84, 86, 87], while 5 (7.9%) reported facilitators to these services [36, 43, 49, 80, 86]. Almost half of the included studies were conducted in the USA (30/63 (47.6%)) [9, 10, 34, 39–41, 45, 53–57, 61, 63, 68, 70–73, 77, 80–83, 85, 86, 88–91], with many others conducted in the UK (27/63 (42.8%)) [21, 23, 25, 35–38, 42–44, 46, 48–50, 52, 58–60, 62, 64–67, 69, 76, 84, 87]; the six remaining studies were conducted in Australia (3/63, 4.7%) [51, 75, 78], Ethiopia (1/63, 1.5%) [74], the Republic of Ireland (1/63, 1.5%) [47] and Singapore (1/63, 1.5%) [79]. Community care was the most common exclusive setting for non-medical prescribing services offered to patients with mental illnesses (43/63, 68.2%) [21, 23, 34, 37–40, 42, 44–48, 50, 51, 53, 54, 56, 60–63, 67–69, 71–73, 75–87, 89, 90], with hospital care alone being a much less popular setting (4/63, 6.3%) [55, 74, 88, 91]. Eleven studies (17.4%) were conducted across both settings [9, 10, 25, 36, 41, 43, 49, 57, 58, 66, 70].

Quantitative designs were commonly used (30/63,47.6%) [34, 36, 39–41, 46, 49–51, 54–56, 61, 63, 65, 67, 70–74, 79–81, 83, 85, 88–91], with 17/63 (26.9%) using a solely qualitative design [9, 21, 23, 35, 37, 42–44, 47, 48, 52, 59, 60, 64, 77, 78, 84] and 16/63 (25.3%) a mixed method [10, 25, 38, 45, 53, 57, 58, 62, 66, 68, 69, 75, 76, 82, 86, 87]. The most commonly reported data collection methods used in the included studies were survey questionnaires (*n* = 36). Among these, 18 studies used a combination of survey questionnaires and other data collection methods [10, 23, 25, 34, 36, 38, 40, 41, 45, 46, 49, 50, 53, 55–58, 62, 63, 65–70, 73, 74, 76, 79, 80, 82, 85–87, 90, 93]. The next most common data gathering technique was interviews (*n* = 21), of which nine utilised interviews alongside other data collection methods [9, 21, 23, 37, 38, 42–44, 47, 48, 52, 58–60, 64, 68, 75–78, 84], followed by claim databases [a well-structured repository where insurance or healthcare billing records document each patient's services, costs, diagnoses, and prescribed medications [94] (*n* = 7, including the Medicare claims database [39, 85], psychotropic drug claims [45], pharmacy claims [54], commercial and Medicare supplemental claims [61], annual computerised administrative claims data [71, 72].

Out of 63 studies, 44 (69.8%) focused entirely on nurse prescribing [9, 21, 23, 25, 34–36, 39, 41, 43–47, 51–60, 64–79, 92], 16 (25.3%) focused on pharmacist prescribing only [10, 38, 40, 42, 48, 62, 63, 81–84, 86, 88–91] and 3 (4.7%) included both professional groups [37, 49, 50]. No other professions were the subject of the included papers apart from nurse and pharmacist prescribers. A summary of the included study characteristics can be found in Table 2.

### Nature of non-medical prescribing services

The nature of NMP services for patients with mental illnesses was separated for nurse and pharmacist NMPs into four categories, organised according to medication/s prescribed, service/s provided, psychiatric disorder/s cared for, and the form of prescribing used in practice (independent prescribing or supervised prescribing). A full summary of the data on nature of NMPs for patients with mental illnesses can be seen in Table 3*.*

**Table 3:** A full summary of the data on the nature of non-medical prescribers for patients with mental illness

The nature of non-medical prescribers for patients with mental illness				
Nurse NMP				
**Country**	**Medication/s reported**	**Service/s provided**	**Psychiatric diagnoses**	**Model of prescribing**
UK	Anti-psychotics [25, 52] Antidepressants [25, 52, 66] Anti-cholinesterase [60] Anxiolytics [25] Hypnotics [25, 66]/ benzodiazepine [66] Methadone (other opiate) [25] Mood stabilizers [25] Anti-dementia [67]	Community Mental Health Team [36]; Assertive Outreach [36, 43], Crisis Intervention Team / Home Treatment [36, 43], Early Intervention [21, 43] Older people Service [25, 36, 43] Perinatal service [43] Substance misuse service [25, 36, 43] Continuing community care [69] Child and adolescent mental health service [46, 87] A neuropsychiatry services [69]	Psychosis [21] ADHD [46, 87] Depression [58] Anxiety [58] Schizophrenia [58] Cognitive impairments [60] Dementia [67]	SP [21, 46, 52, 58, 60, 87] IP [67, 69, 76] IP/SP [25, 36, 43, 65]
USA	Antidepressants [34, 45, 71, 72]: SSRIs [55], SNRIs [56] Anti-convulsant [45, 71, 72] Mood stabilizers [45, 70]: lithium [71, 72] Anti-psychotics [45, 71, 72, 80] CNS stimulants [45, 56]: Controlled and non-controlled substances for ADHD [54], medications for ADHD [70–72], LA and SA stimulants [73] Sedatives & hypnotics [45, 70–72] Anxiolytics [55, 70–72] Anti- cholinesterase: Donepezil, galantamine, rivastigmine, memantine, combination donepezil and memantine [61] Medication for PSTD, schizophrenia, SUD, mania [70] Alpha-agonists [71, 72] Psychotropic medications [77]	Outpatient and psychotherapy service [39] Crisis Intervention Team [41] Child and adolescent psychotherapy/ psychiatry [41, 53, 72] Individual/family psychotherapy [41] Prescribing medication management [41] Diagnostic evaluation [41] Psychoeducation [41] Case management [41] Consultation or liaison [41] Behaviour health service [45] Assertive Outreach [68] Service for nursing home residents [85]	SUD [34, 39] Disruptive behaviour disorder [34] Anxiety [34, 39, 55, 70–72] Bipolar [39, 85] Depression [39, 55, 70–72, 85]/MDD [45] Schizophrenia [39, 70, 85] Psychosis [39] AUD [39] ASD [39] Trauma [41] Eating disorder [70] Bereavement/grief [41] Personality disorder [41, 70, 85] Neurocognitive disorder [41] ADHD [54–56, 73] Difficulty coping with psychological distress [55] Alzheimer disease [61, 70], ADRD [85] Dementia [70] Mania [70] PSTD [70] Drug use disorder [85]	SP [57, 68, 77] IP [61, 71] IP/SP [9, 34, 39, 85, 95]
Australia	Antidepressants [75] Benzodiazepines [75]	The Crisis, Assessment and Treatment Service [78] Perinatal mental health service [51]	Depression [51, 75] Anxiety [51, 75] Adjustment disorder [51] Substance misuse [51] Mild mental retardation [51]	SP [51, 75, 78]
Singapore	NS	NS	Schizophrenia/depression/bipolar disorder/ anxiety [79]	SP [79]
Ethiopia	NS	NS	Common psychiatric disorders [74]	SP [74]
Pharmacist NMPs				
**Country**	**Medication/s reported**	**Service/s provided**	**Psychiatric diagnoses**	**Model of prescribing**
UK	Antidepressants: sertraline, fluoxetine, mirtazapine, citalopram, duloxetine [38] Benzodiazepines: Zopiclone, diazepam [38] ADHD medications [62] Z-drugs (zopiclone, zolpidem and zaleplon) [84]	Community team learning disability service [48] Child and adolescent mental health services [62]	Mixed depression and anxiety [38] Depression [26, 38] Anxiety [38] Low mood related to bereavement [38] Emotionally unstable personality disorder [38] PSTD [38] Bipolar disorder [26] Psychosis [26] Learning disability and behaviours disorder [48] ADHD [62] Acute insomnia [84]	IP [48, 62] SP [48]
US	Antidepressants [81]: mirtazapine, fluoxetine, amitriptyline, citalopram [40] Anti-psychotics [81, 83]: quetiapine [88, 91], ziprasidone [40] Psychotropic medication [63, 83, 89] CNS stimulants [81]: amphetamine and amphetamine-like stimulants [10], benzodiazepines [10] [81] Z-drugs [81] Antianxiety [81] Meds for EPS [81] Mood stablisers [81] Other sleep aids [81] Clozapine [86]	Psychiatric emergency services (PES) [81] The Veterans Integrated Services Network [82] Home Treatment [83] Clozapine clinic [86]	Major depressive disorder [40, 89] depressive disorder [81, 90], Unspecified depression [89], Major depressive disorder with psychosis [89] Schizo affective disorder [40, 89] Panic disorder [40, 89] ETOH/substance abuse /dependence [40] Alcohol use [82, 89], Nicotine use [82], Cannabis use [82] Bipolar disorder [40, 81, 82, 90], Bipolar I [89], Bipolar II [89], Bipolar with psychosis [89] Schizophrenia [40, 82, 89] Treatment resistant schizophrenia [86] Obsessive- compulsive disorder [40, 89] ADHD [40, 81, 82] Generalized anxiety [40] Depression [63, 82] PSTD [40, 81, 82, 89] Anxiety disorder [81, 82, 90], Generalized anxiety disorder [89] Eating disorder [81] Personality disorder [81] Psychotic disorder [81], Psychosis not otherwise specified [89] Sleep disorder [81], Insomnia [82] Dementia with behavioural disturbance [83] ICU delirium [88, 91] Unspecified mood disorder [89] Intermittent explosive [89]	SP [63, 83, 86, 88, 90, 91] IP [10, 81, 82, 89]
Both NMPs				
**Country**	**Medication/s reported**	**Service/s provided**	**Psychiatric diagnoses**	**Model of prescribing**
UK	Benzodiazepines [37] Anti-psychotics (atypical, typical) [50] Antidepressants [50] Hypnotics [50] Anxiolytics [50] Mood stabilizers: lithium, valproate [50] Stimulants [50] Medication for opiate dependency [50] Anti-dementia [50]	Forensic service [49] Assertive Outreach [49, 50] Child and adolescent mental health service [49, 50] Home Treatment [50] Early Intervention [50] Acute inpatient epilepsy psychiatric intensive care [49] Community team learning disability [49] Mental health service for old people Drug and Alcohol Team [50] General adult psychiatry [50]	Psychotic illness [50] Affective illness [50] Insomnia [50] Extra-pyramidal side effect [50] Anxiety disorder [50]	IP [37] SP [49] IP/SP [50]

*ADHD* Attention-Deficit/Hyperactivity Disorder, *SP* Supervised Prescribing, *IP* Independent Prescribing, *SSRIs* Selective Serotonin Reuptake Inhibitors, *SNRIs* Serotonin-Norepinephrine Reuptake Inhibitors, *SUD* Substance Use Disorder, *MDD* Major Depression Disorder, *CNS* Central Nervous System, *LA* Long Acting, *SA* Short Acting, *PSTD* Post-Stress Traumatic Disorder, *AUD* Alcohol Use Disorder, *ASD* Autism Spectrum Disorder, *ETOH* Ethyl Alcohol, NS: Not stated

### Nature of nurse non-medical prescribing

#### Medications prescribed by nurse non-medical prescribers 

In the UK, slightly over one-third of studies (5/14, 35.7 reported on which medications nurses were prescribing for patients with mental illnesses [21, 46, 58, 60, 67]. Of these, antidepressants were the most commonly listed (60%) [25, 52, 66], followed by antipsychotics (40%) [25, 52].

In the USA, out of 19 studies, 11 (57.8%) reported which medication/class of psychotropic medication nurses were prescribing and managing [34, 45, 54–56, 61, 70–73, 77], with central nervous system (CNS) stimulants being reported in use by almost two-thirds of the studies (7/11, 63.6%), mainly for attention deficit hyperactivity disorder (ADHD) [45, 54, 56, 70–73]. Antidepressants (6/11, 54.5%) were the second most reported class of medicines [34, 45, 55, 56, 71, 72].

Antidepressants and benzodiazepines were reported in one study out of three conducted in Australia (33.3%) [75]. No information was provided on medications prescribed by nurses in studies conducted in Singapore and Ethiopia.

#### Services nurse non-medical prescribers provided

Half of the studies based in the UK (7/14, 50%) reported the services nurse NMPs were working in [21, 25, 36, 43, 46, 69, 87]. Of these, services for older people (3/7, 42.8%) [25, 36, 43] and drug/alcohol services (3/7, 42.8%) [25, 36, 43] were reported.

In the USA, a total of seven studies out of 19 (36.8%) described the nature of NMP services [39, 41, 45, 53, 68, 72, 85]. Of these, child and adolescent mental health services were mentioned in three studies (42.8%) making these the most commonly reported NMP services [41, 53, 72].

A crisis, assessment, treatment (CAT) service and specialist perinatal mental health service were each reported once in the Australian studies (2/3, 66.6%) [51, 78]. No information was provided on the types of services provided in Singapore and Ethiopia.

#### Patients with psychiatric disorders cared for by nurse non-medical prescribers

In the UK, over one-third of the studies (5/14, 35.7%) included which mental health illnesses nurse prescribers managed [21, 46, 58, 60, 67]. Of these studies, seven different diagnoses were described, including cognitive disorders (e.g., cognitive impairment, dementia), which featured in 40% percent of the studies (2/5), making them among the most common conditions listed [60, 67].

In the USA, the majority of studies (12/19, 63.1%) described which psychiatric condition(s) the patients had been diagnosed with [34, 39, 41, 54–56, 61, 70–73, 95]. There were 20 diagnoses described, with anxiety and depression reported by the majority of studies (7/12, 58.3%) [34, 39, 55, 70–72, 95], and around a further third reporting ADHD (4/12, 33.3%) [54–56, 73].

In studies conducted in Australia, patients with depression and anxiety were the focus of two out of three studies (66.6%) [51, 75]. In the Singapore study, schizophrenia, depression, bipolar disorder, and anxiety were reported [79].

#### The model of prescribing for nurse non-medical prescribers

All except one of the studies which was carried out in the UK reported which form of prescribing nurse NMPs were practising (13/14, 92.8%) [21, 25, 36, 43, 46, 52, 58, 60, 65, 67, 69, 76, 87]. Of these, 6/13 (46.1%) were practising with some form of supervision (supervised prescribing); [21, 46, 52, 58, 60, 87] practising with independent authority was reported in less than one-quarter (23%) of the studies (3/13) [67, 69, 76]. Practising under supervision and independently were both studied together in four studies (30.7%) [25, 36, 43, 65].

In the USA, nearly half of the studies (9/19, 47.3%) reported which form of prescribing nurse NMPs were practising [9, 34, 39, 57, 61, 68, 71, 77, 95]. While the two forms of prescribing (independent and supervised) were studied together in four studies (4/9, 44.4%) [9, 34, 39, 95]. Of the remaining five studies, three (3/9, 33.3%) reported practising only under supervision [57, 68, 77], and two (22.2%) recent studies examined those practising independently [61, 71].

Nurse NMPs in all other studies, from Australia [51, 75, 78], Singapore [79], and Ethiopia, [74] were reported to be practising under supervision.

### Nature of pharmacist non-medical prescribing

#### Medications prescribed by pharmacist non-medical prescribers

Among the five UK studies, only three (60%) reported which medication pharmacists were prescribing. Among these, three drug classes were identified; antidepressants such as sertraline, fluoxetine, mirtazapine, venlafaxine, duloxetine, and citalopram [38]; hypnotics such as zopiclone and diazepam [38, 84]; and medications for ADHD [62]. In the USA, out of 11 studies, nine (81.8%) reported which medications pharmacists were prescribing. The antipsychotics class of medications (e.g., quetiapine [88, 91], and ziprasidone [40] was the most commonly listed in the USA-based studies (4/9, 44.4%) [81, 83].

#### Services provided by pharmacist non-medical prescribers

Only two UK studies out of five reported which services pharmacist NMPs were providing (40%) [48, 62]. Community team learning disability [48] and child/adolescent mental health services [62] were reported in this context. In USA-based studies, only five reported which service pharmacists were practising in (5/11, 45.4%). Among these, services for intensive care unit (ICU) patients with delirium were mentioned twice [88, 91].

#### Patients with psychiatric disorders cared for by pharmacist non-medical prescribers

All five UK based studies [38, 42, 48, 62, 84], and all but one study in the USA [40, 63, 81–83, 86, 88–91] (reporting rates of 100% and 90.9, respectively) reported the types of mental illnesses patients had while being cared for by pharmacist NMPs. Among the UK studies, depression was the most commonly reported mental illness, which was mentioned in two studies (40%) [38, 42]. Bipolar disorders were listed in just under half of the USA-based studies (5/11, 45.4%) [40, 81, 82, 89, 90], with depression [40, 81, 89, 90] and anxiety [81, 82, 89, 90] tied as the second most commonly listed issues (4/11, 36.3%).

#### The model of prescribing for pharmacist non-medical prescribers

Prescribing independently was reported as the practice model by most of the UK studies (3/5) [38, 48, 62]. The practice model was described in all except one USA-based study (10/11, 90.9%), with practising under supervision accounting for over half of these (6/10, 60%) [63, 83, 86, 88, 90, 91].

### Studies exploring the nature of non-medical prescribing by nurses and pharmacists

All data from studies exploring the nature of NMP for nurses and pharmacists together came from the UK (3/3, 100%) [37, 49, 50]. Of those, two studies (66.6%) reported on the medication NMPs were prescribing, with medications reported once in each study: these were benzodiazepines [37], medications for opiate dependency [50], and antidepressants (SSRI, TCA, and other) [50] (33.3% each). Services for children and adolescents and assertive outreach were reported by the two studies that provided data on service type (2/2, 100%) [49, 50]. One of the studies reported which mental health diagnosis patients had received, mentioning psychotic/affective illness (1/3, 33.3%) [50]. Pharmacists and nurses were practising as independent prescribers in one study [37], and under supervision in another study [49]. Both models of practice were studied by the remaining study (33.3%) [50].

#### The impact of non-medical prescribing to patients with mental illnesses

A total of 45/63 (71.4%) studies reported data on the impact of NMP [21, 23, 25, 35, 38, 40, 42–44, 46–53, 57–60, 62–69, 74–79, 81–83, 85–91]. Data on the impact of NMP for patients with mental illnesses were measured on four levels: these were the effects on NMPs themselves (*n* = 8), on patients managed by NMPs (*n* = 39), on other healthcare professionals (*n* = 11), and on the wider healthcare system (*n* = 5). Overall, the majority of the impact data originated from studies conducted in the UK (24/45, 53.3%). A full summary of the data regarding the impact of NMP to patients with mental illnesses can be viewed in Table 4.

**Table 4:** A full summary of the data of the impact of non-medical prescribing for patients with mental illness

The impact of non-medical prescribing			
	Nurse NMPs	Pharmacists NMPs	Both NMPs (nurse, pharmacist)
On NMPs themselves	Increase insight into holistic care [23, 35, 59] NMP’s value increased when sought [21, 35] Increase the support from colleagues [35] Increase the ability to monitor SE [21]/medication [57] Increase looking up to interactions [65] Involve more in different activities (e.g., PGD) [66] Increase reviewing medication to reduce inappropriate prescribing and polypharmacy [23] Further enhance status of nursing [57] Increase the public’s awareness of nursing [57] Increase the ability to fulfil patients need [21] The level of confidence increased [21] (Feel more empowered [23, 57], and increase the prestige) [57] Intellectual stimulating [57] Level of knowledge increased [66] High level of job satisfaction [25, 57]	More empowered [48]	Further enhance status of nursing [49] Increase the legitimateness [49] Increase the opportunities for nurses to lead a clinic and service [49] Workload increased [50] Increase the ability to monitor SE [49] Level of confidence/ job satisfaction increased [49] More knowledgeable [49] Receive more respect [49]
On Patients with mental illness	Improve the therapeutic relationship [21, 23, 47, 60, 76] High satisfaction with the service and the treatment [58, 60, 69, 76, 78, 79, 87] Increase knowledge and education [21, 57, 59, 67, 78] The continuity of care increased [21, 35] Condition well managed: reduction each of depression, behaviour and psychiatric symptoms [51, 79], fast recovery [79], reduced the hospitalisation [75] NMPs more convenient: increased the accessibility [21, 53, 60, 64, 69, 76, 78], home-based treatment [47, 78] Provide holistic care [47, 52, 60, 77] Involve more in decision making process [21, 23, 47, 76, 87]: improve the adherence/concordance [21, 23, 47, 60, 79] Improved the communication [65, 77, 78] Reduction of waiting time [53, 60] Feeling less stress with nurse NMPs [21] No difference in each of (adherence/mental health/SE/ satisfaction with overall care) [58],no significant difference in each of (SDQ for ADHD, SDQ for CD, SDQ for ED), (satisfaction) and (side effect) [46] Less satisfaction level with nurse NMPs [68] Increased risk of hospitalisation [58] Lower hospitalization rates with NPs [85] Not fully satisfied (e.g., missing appointment notes and difficulties in getting new medications) [87]	The therapeutic relationship improved [38, 42] High satisfaction with the service and the treatment [40] Becoming more knowledgeable [42, 62] The follow up care improved [42] Condition well managed: reduced depression [63]/generalized anxiety symptoms [38], low number of failures [63] More convenient and increased the access [42, 63, 81, 82] Decision made in partnership [42, 48] Waiting time reduced [63] Improved the consultation [48, 62] Improved patients’ outcome (decreased total medications, reduced ACB Scale, decreased psychotropic medications) [83] Stopped inappropriate medications [83] reduced inappropriate medications [88] Reduction in antipsychotic continuation at hospital discharge (*P* < *.001*) [91] Reduction unnecessary antipsychotic use in the ICU once delirium resolved (*P* = *.015*) [91] No increase in length of stay at hospital [91] No statistically significant in reoccurrence of ICU delirium after antipsychotic discontinuation (*P* = *.236*) [91] No statistically significant differences in various patient outcomes (such as A1c, BMI, weight, cholesterol levels, blood pressure, medication use, and clozapine dose) [86] Patients preferred Psychiatrist (more appointment attended) [89] High medication adherence [89] Up-to-Date laboratory monitoring [89]	Improved the access [49, 50]
On other healthcare professionals	Increase the communication and collaboration [43, 44, 68, 77] Increase the multidisciplinary approach [43] Reduce doctor workload [44] Increase the team knowledge [69] Positive effect of the MHT’s skills [52] Increased the confident in staff to manage end of life related issues [75]	Improve the collaboration [48] MDT receive more support [48] Improve medication debate [48] Reduced the anxiety in workplace [62] (Gain Knowledge [90] and broader perspectives) [48] Increased confidence in psychotropic’s prescribing [90]. The work environment improved after having clinical pharmacist [90].	The multi-professional functioning improved [49]
On the healthcare system	Cost-effective service [53, 57, 77] Increased the capacity [53]: doubled [77] It improved the connection between each of SPC, GPs, RACFs [75] The delivery of healthcare was improved [64]	NS	NS
Other (not specified)	The service was rated as excellent by medical collages [74]	NS	NS

*NS* Not Stated, *PGD* Patient Group Direction, *SE* Side Effect, *NMPs* Non-Medical Prescribers, *MHT’s* Mental Health Teams, *MDT* Multidisciplinary Team, *SPC* Specialist Palliative Care, *GPs* General Practitioners, *RACFs*: Residential Aged Care Facilities’, *SDQ* the Strengths and Difficulties Questionnaire, *ADHD*, Attention Deficit Hyperactivity Disorder, *CD* Conduct Disorder, *ED* Emotional Disorder

### Impact on nurse/pharmacists’ prescribers themselves

#### On nurse non-medical prescribers

Studies from the UK provided almost all data on the impact of NMP on nurse prescribers themselves (7 studies out of 8 studies, 87.5%) [21, 23, 25, 35, 59, 65, 66], with one such study originating from the USA (1/8, 12.5%) [57]. Nurse prescribers in three qualitative studies described how their ability to provide a holistic patient-centred approach improved after they qualified as prescribers (3/8, 37.5%) [23, 35, 59]. A quarter of the studies (2/8) used questionnaires and focus groups [25, 57], and these all mentioned job satisfaction being increased once nurses became prescribers. A full summary of the data is offered in Table 4*.*

#### On pharmacist non-medical prescribers

Data concerning the impact of NMP services on pharmacist prescribers came from one study, carried out in the UK (1/1, 100%) [48]. Those participants felt empowered as prescribers, believing they could better address medication use for off-label conditions.

#### Both nurse and pharmacist non-medical prescribers

Two UK based studies, which used questionnaire designs, measured the impact of NMP services on both nurses and pharmacists [49, 50]. They linked job satisfaction and increased confidence to prescribing authority, with one connecting it to developing independent prescribing [49]. Both nurse and pharmacist prescribers mentioned significantly increased workloads as a result of their prescribing responsibilities [50].

### Impact on patients with mental illness managed by nurse/ or pharmacist non-medical prescribers

#### Patients managed by nurse non-medical prescribers

A total of 24 studies out of 39 (61.5%) studies that measured impact on patients with mental illness focused on patients managed by nurse prescribers [21, 23, 35, 46, 47, 51–53, 57–60, 64, 65, 67–69, 75–79, 85, 87]. More than half of these studies were from the UK (14/24, 58.3%) [21, 23, 35, 46, 52, 58–60, 64, 65, 67, 69, 76, 87], followed by the USA (5/24, 20.8%) [53, 57, 68, 77].

Seven studies out of twenty-four (29.1%) measured patient satisfaction by using a mixed qualitative and quantitative study design. These studies reported that patients were satisfied with the services and the treatment received, perceiving receipt of effective services from nurse prescribers [58, 60, 69, 76, 78, 79, 87]. Patients also reported nurse NMP services as being more convenient in over a third of the studies (8/24, 33.3%), as they were seen as more accessible; home-based treatment was also acknowledged as being convenient [21, 47, 53, 60, 64, 69, 76, 78]. The sole Australian study (1/24, 4.1%) focusing on mental health services delivered to perinatal women observed a reduction in depression symptom scores based on the Edinburgh Depression Scale (EDS), with a score reduction from 18 to 10 (*p* < 0.001) [51]. The same study noted an improvement in behavioural functioning among patients cared for by the nurse NMPs [51].

One study (4.1%) conducted in the UK reported that patients were more satisfied with psychiatrists than nurse prescribers, as they found psychiatrists were more accessible, spent sufficient time with them, and discussed their symptoms [68].

#### Patients managed by pharmacist non-medical prescribers

Only thirteen studies out of 39 (33.3%) included impact data on patients managed by pharmacists [38, 40, 42, 48, 62, 63, 81–83, 86, 88, 89, 91]; of these, the majority were USA-based (9/13, 69.2%) [40, 63, 81–83, 86, 88, 89, 91], with four studies (30.7%) conducted in the UK [38, 42, 48, 62].

One study gathered views from patients managed by pharmacist NMPs using a questionnaire; this reported that satisfaction was high, as the pharmacists met their patients’ needs by providing high quality services [40]. Patients reported improved access to pharmacist non-medical prescribing services when compared to doctors in two studies [42, 63]. Two UK and USA based studies that focused on services provided by mental health specialist pharmacists in primary care/general practice reported reductions of 50% in depression/anxiety symptom scores based on the Patient Health Questionnaire (PHQ-9) and Generalised Anxiety Disorder (GAD-7) [38], and in excess of a 50% reduction in depression score based on PHQ-9) [63]. One study quantitatively measured the waiting times for patients managed by psychiatric pharmacists compared to those seen by behavioural clinic providers, reporting that the waiting time for treatment initiation for patients was three times less with pharmacists than with behavioural clinic providers (on average, 22.6 days versus 79.3 days, respectively) [63]. The prescribing of inappropriate medications was also reduced and, in many cases, ceased [83, 88]. Unnecessary antipsychotic prescribing was particularly reduced (*P* = 0.015), [91] based on quantitative observations in studies conducted in the USA.

#### Patients managed by both prescribers

Two (2/39, 5.1%) studies conducted in the UK included patients with mental illness managed by both nurse and pharmacist NMPs [49, 50]. These studies found that increased prescriber availability improved access to services and medications, especially in isolated areas [49, 50].

### Impact of non-medical prescribing on other healthcare professionals

#### Nurse non-medical prescribers

The majority of studies (7/11, 63.6%) reporting on the impact of NMP on healthcare professionals focussed on nurse NMPs; of these, four were based in the UK, two in the USA and one in Australia [43, 44, 52, 68, 69, 75, 77]. Over half (4/7, 57.1%) reported communication with the team and multidisciplinary approaches as being improved with nurse prescriber involvement [43, 44, 68, 77]. This improvement was noted by nurse prescribers [43], team members [68], a nursing administrator [77], and psychiatrists and healthcare professionals [44], within interviews, [43, 77], surveys [68], and interview and focus group work [44]. Three other studies mentioned that the team’s knowledge [69] and skills [52], as well as staff confidence [75] increased following the involvement of nurse prescribers. A full summary of the data is given in Table 4*.*

#### Pharmacist non-medical prescribers

Over a quarter of the studies (3/11, 27.2%) reported data concerning the impact of NMP on healthcare professionals focused on pharmacists; all but one of these (USA) [90] were conducted in the UK [48, 62]. One study (33.3%) noted that pharmacist prescribers enhanced shared care and collaborative discussions on medication, according to staff interviews [48]. A full summary of this data is given in Table 4*.*

#### Both nurse and pharmacist non-medical prescribers

One study (9%) conducted in the UK included both types of NMPs (nurse and pharmacist). This reported data concerning the impact of NMP on healthcare professionals more generally, noting that NMP enhanced healthcare professionals' work and collaboration [49].

### The impact of non-medical prescribing on the healthcare system

Only five studies (5/45, 11.1%) measured the impact of NMP on the wider healthcare system; all of these were focused on nurse non-medical prescribing [53, 57, 64, 75, 77]. The capacity of healthcare services being increased was reported in two studies from the USA, [53, 77] with one medical director reporting that capacity to deliver service was doubled. Three studies from the USA also reported that child and adolescent services provided by nurse NMPs were more cost-effective compared to psychiatrist services. This was reported to be due to enhancement in access to healthcare, which especially benefited those previously under-served by the healthcare system [57]. A study reported that Psychiatric Mental Health Nurse Practitioners (PMHNPs) were more cost-effective than psychiatrists, saving $10,000 to $20,000 annually, according to mental health directors’ estimations in two U.S. counties [77].

#### Factors influencing implementation and delivery of non-medical prescribing services

Sixteen out of sixty-three (25.3%) studies identified factors influencing the successful implementation and delivery of NMP services [9, 21, 25, 36–38, 43, 49, 52, 59, 64, 74, 80, 84, 86, 87]. Fourteen (22.2%) reported data on barriers [9, 21, 25, 36–38, 52, 59, 64, 74, 80, 84, 86, 87], and five studies (7.9%) discussed facilitators to NMP services [36, 43, 49, 80, 86]. Most data on barriers originated from studies conducted in the UK (9/14, 64.24%), followed by those from the USA (4/14, 28.5%), and Ethiopia (1/14, 7.1%). Three studies that reported facilitators originated from the UK (3/5, 60%), with two from the USA (2/5, 40%). A full summary of the data is given in Table 5*.*

**Table 5:** A full summary of the data on the factors influencing the successful implementation and/or delivery of non-medical prescribing services

	Barriers to implementing and/or delivering non-medical prescribing services	
**NMP type**	**Country**	
Nurse	UK	• Lack of the appropriate training and time [21, 52] • Lack of medical support/ supervision [25, 36] • Hard to meet the criteria for nursing prescribing programme [36] • No clear structure [21] (e.g., policies and clinical governance) [25] and absence of well-defined approach to implement [36] • Funding relating to training programme [36] • Lack of financial remuneration [21, 25] • Unclarity around the role of NMPs (job description) [21, 25] • Restriction with supplementary prescribing [59] • The reaction of medical colleagues towards nurse prescribing (doctor threatened by nurse being prescriber) [59] • Fears of re-located from community to more specialised clinic [59] • Nurse prescriber not seen competent as medical prescriber [59] • Inability to access to prescription pads [25] • Lack of communication and information dissemination [25] • Prescribing from limited formulary [64] • Shortage of staff [87] • Issue with Continuing Professional Development [87] • Not able to deal with case complexity thoroughly due to lack the time [87].
	USA	• Supervision related issue [9] • Low salary and incentives [77] • Nurse prescriber not seen competent as medical prescriber [77] • Lack of confidence in managing the risk associated with antipsychotics medications [80].
	Ethiopia	• Low salary [74] • Absence of the essential drug in place [74]
**NMP type**	**Country**	
Pharmacist	UK	• Constraints of the space in practice [38] • Difficulties in accessing both electronic health records and prescription [38] • lack of understanding of pharmacist role (from patients) [84] • long-time taking in reviewing Z-Drug (e.g., a class of medicines that are primarily used as sedative-hypnotics) [84]
	USA	• Difficulty associated with timely collection and reporting of Absolute Neutrophil Counts (ANC) to the pharmacy [86]. • Concern related to tolerability and side effects of clozapine [86]. • Inadequate knowledge in managing clozapine side effects and clinic procedure [86]. • Lack of written materials for patient education and lack of experience with clozapine prescribing [86]. • Not fully familiar with clozapine prescribing guidelines [86].
Both	UK	• The difficulty associated with the electronic system [37] • Lack of the appropriate training and time (with benzodiazepine prescription) [37] • No clear structure of CPD [37] • Lack of support and supervision [37]
	Facilitators to implementing and/or delivering non-medical prescribing services	
**NMP type**	**Country**	
Nurse	UK	• Availability of a lead person to support nurse prescribing practice [36] • Availability of defined formularies in place [43]
	USA	• Educational activities for continuing education (CE) Credits [80]. • Reading both journal articles and textbooks [80]. • Consulting with specialists [80]. • Attending conferences [80]. • Content in NP program [80]. • Having a pharmaceutical representative [80].
Pharmacist	USA	• Having ongoing clozapine education [86]. • Adequate administrative support (for managing the REMS registry and initiating clozapine) [86].
Both	UK	• Availability of several guidance and publications sources [49].

*NA* Not Available, *CPD* Clinical Professional Development, *NMPs* Non-Medical Prescribers

Almost half of the studies conducted in the UK (4/9, 44.4%) reported training-related issues as being a barrier to NMP services in both the implementation and delivery phases [21, 36, 37, 52]. Issues included funding related to training programmes [36], inadequate training in mental health due to NMP courses being overly generic, [21, 52] and limited training in the use of benzodiazepines [37]. A lack of clarity regarding the structures, policies, and clinical governance frameworks supporting NMP, combined with an absence of defined approach to implementation, was also identified as a barrier in several UK-based studies. (3/9, 33.3%) [21, 25, 36]. Not receiving adequate support from medical professionals was further reported as a barrier to service implementation in some UK studies (3/9, 33.3%) [25, 36, 37].

In studies conducted in the USA, nurse prescribers mentioned financial barriers (2/4, 50%) including high supervision fees [9] and their low salaries as compared to medical professionals [77]. Pharmacists in another study raised the issue with having insufficient knowledge to deal with clozapine related side effects [86].

In terms of facilitators, one UK study (1/5, 20%) reported that the availability of support from a lead person was a facilitator for NMP practices [36]. Having defined formularies in place (1/5, 20%) [43], and the ready availability of different guidelines (e.g., Nursing and Midwifery Council standards; National Prescribing Centre publications and website; NIMHE/ Department of Health guidance; peer group information networks) and other valuable resources (e.g., the British National Formulary) (1/5, 20%) [49] were also reported as facilitators for service implementation by both nurse and pharmacist NMPs.

## Discussion

To our knowledge, this scoping review is the first comprehensive study to collate contemporary global evidence focusing on the nature, impact, and factors influencing the successful implementation and delivery of NMP for patients with mental illnesses across healthcare settings.

In total, 63 studies were identified, of which 57 reported on the nature of NMP services, 45 examined service impact, and 16 explored factors influencing service implementation and/or delivery. Most of the studies (*n* = 44, 69.8%) were solely focused on nurse NMPs, with comparatively fewer featuring pharmacist NMPs (*n* = 16). Only three included both nurse and pharmacist prescribers.

With regard to the nature of nursing NMP services, antidepressants and CNS stimulants were among the most commonly prescribed medications, leading in the UK and the USA, respectively. Patients managed by nurse and pharmacist NMPs generally expressed high levels of satisfaction with the service provided. In the UK, the most frequently mentioned barriers were associated with training, whereas financial issues were reported in the USA. The findings that emerge from this study may thus be used by health bodies, societies, and regulators to advance policy and practice regarding NMP services for patients with mental illnesses, particularly in these countries.

In this review, limited numbers of studies reported on which medications NMPs were prescribing. However, among those which did provide this information, certain patterns emerged. In both the USA and the UK, where most of the data came from, antidepressants were the class of medications most frequently prescribed by nurse NMPs, being the most prominent overall in the UK, and second in the USA. Recent statistics reported in both the UK and USA show that antidepressant medications are commonly prescribed [96–100]. However, the appropriateness of antidepressant prescribing has been questioned, particularly in the longer term [101, 102]. Further research should thus explore the reasons behind the prescribing choices made by nurse NMPs in this regard. CNS stimulants were reported as being prescribed by NMPs in both the UK and USA. In USA-based studies, these medications were explicitly reported for ADHD treatment. However, most studies did not specify whether the prescriptions were made in general practice or within dedicated ADHD services, making it unclear how widely these medications were prescribed outside of ADHD-focused care. Providing such information would thus be beneficial to further analysis. Recent reports and studies indicate a growing disparity between the increasing rate of ADHD diagnosis and limitations in supply of these medications at national level [103–107]. NMPs may therefore play an important role in addressing these challenges by providing appropriate and timely access to treatment, including alternative options, and further research should build on the findings of this review to explore this in more detail. While pharmacists in the USA-based studies were mainly involved with anti-psychotic medications, their role focused on deprescribing inappropriate medications that patients were already taking rather than prescribing further medications. A systematic review of 24 studies published in 2021 which aimed to examine the impact of pharmacist-led deprescribing highlighted the importance of having pharmacists, who, as medication experts, could thoroughly review patients' medications to assess their appropriateness and discontinue those that were no longer necessary, which can effectively reduce both the number of medications and their side effects [108].

This review noted variation in prescribing models among NMPs across countries, with prescribing autonomously seen more commonly in the UK than the USA. Based on a report published in 2024 that compared the UK, the USA, and Australia, NMPs, and pharmacists in particular, were noted to have more restricted authority regarding which medications or conditions they could manage in the USA and Australia when compared to the UK [109]. This indicates that the UK has made more progress than other countries in terms of expanding prescriptive authority to NMPs. However, in the studies included in this scoping review, the reasons behind the UK's move toward independent prescribing are not clearly explained as this may not have been their primary focus. NHS Long Term Workforce plan identifies factors such as workforce shortages, increasing healthcare demands, and efforts to improve patient access to medicines as key contributors to the need to address workforce challenges and support the shift towards independent prescribing [5].

In this review, the impact of NMP for patients with mental illnesses can be seen to extend across multiple dimensions, significantly benefiting patients, providers, healthcare professionals, and the wider healthcare system. Nurse NMPs in this review perceived the prescribing authority as enhancing their ability to provide a more holistic care approach to patients, in alignment with Bradley et al., who found similar results in an interview study conducted in the UK in 2007 with nurse NMPs practising in both acute and community sectors [110]. While the existing evidence did not clearly demonstrate that medical prescribers can provide holistic care to mental health patients or provide a comparison between the two professional groups, this review supports the idea that nurse NMPs can provide more holistic and patient-centred care. This has the potential to have a positive impact on prescribing, given the complexities and comorbidities that mental health patients often have. Carey et al. linked providing comprehensive care to prescribing autonomously, as this allows nurses prescribing for patients with respiratory conditions to integrate medication and more comprehensive care in a manner that offers them increased job satisfaction [111]. Enhanced job satisfaction was not limited to independent prescribing; increases were also seen with minimal prescriptive authority (e.g., prescribing under supervision), as this was still linked to greater clinical responsibility. This view was supported by a study included in this review in which job satisfaction was mentioned while prescribing under supervision [25]. While NMP was seen to be generally beneficial for NMP staff, one study in this review did report increased workloads for both nurse and pharmacist NMPs, with this being associated with expanding their responsibilities. However, that study did not clarify whether prescribing duties were seen as an additional responsibility added to their existing role, which might thus explain the increase in workload. This issue may require further investigation in the mental health context, as increased workloads have been identified as a barrier for NMPs with regard to delivering high-quality services across UK healthcare settings [20]. Workload may potentially increase for prescribers caring for mental health patients, as mental health care is often associated with complex, ongoing management, including the monitoring and adjustment of treatment plans, all of which add extra responsibilities [112, 113].

As Woolhouse et al. suggested, several benefits arose from the introduction of NMP in clozapine clinics in 2009, such as more rapid access to medication and services, the provision of more effective services, and the enhancement of the therapeutic relationship [114]. This aligns closely with the results reported in this review for patients with mental illnesses, and with those reported by other patient groups in research studies outside of mental health settings [18, 115–117]. In this review, the impact of pharmacists in terms of improvements in the health of mental health patients was more evident in USA-based studies than those from the UK, highlighting pharmacists’ ability to reduce the inappropriate medications common in the former country. However, it is important to note that most of these pharmacists were working under supervision, with specific collaborative practice agreements. As most of the included studies that evaluated the impact in this review were qualitative in nature, there is also a pressing need for quantitative measurement at scale to evaluate various clinical outcomes, such as clinical appropriateness and safety of medications and rates of adherence.

As several studies outside of the mental health context have previously observed, inadequate education and training, lack of support and supervision, a lack of clear structure, and being under-rewarded for their work were consistently identified as significant challenges to NMP staff providing non-medical prescribing services in this review [118–121]. While education and training were not mentioned as barriers in the studies from the USA, this may be due to the limited number of studies (*n* = 4). The requirements to qualify as a prescriber also vary across countries. In the USA, for instance, nurses must complete an advanced-level education programme to prescribe, whereas in the UK, non-medical prescribers can undertake a programme separately from any advanced-level training. This difference may explain why limited training was identified as a barrier in the UK but not in the USA.

While medical prescribers viewed nurse NMPs as a cost-effective alternative to psychiatrists in this review, NMPs themselves frequently mention feeling under-rewarded and unrecognised. These contrasting views prompt an important question regarding the potential gap between the perceived cost-effectiveness services of NMP and the actual recognition and compensation of the professionals who fulfil these roles. The disconnect between these two views is a critical point of exploration, as work-related stress might lead to burnout, as noted by some nurse NMPs, based on increased pressure and responsibilities without adequate compensation [120]. However, cost-effectiveness was only measured qualitatively in interviews or based on estimations in this review, indicating the need for further quantitative evaluation to determine the cost-effectiveness of the services provided by NMPs as compared to those offered by medical staff more accurately.

In this review, only 16 studies focused solely on pharmacist NMPs (12 USA, 4 UK) resulting in an evidence gap as compared to the data on nurse NMPs. Most of these studies also primarily addressed the nature and the impact of pharmacist prescribing, with very few focused on the influencing factors. This might be due to the longer history of nurse prescribing as compared to pharmacist prescribing: studies including nurse NMPs have been published since 2004, whereas pharmacist focused studies were first identified from 2008. Nonetheless, studies support the positive role of pharmacist prescribers in terms of managing patients with chronic disease in therapeutic areas other than mental health [18, 122], highlighting the need for more research into pharmacist prescribing for patients with mental illnesses, particularly regarding factors influencing implementation and delivery, as the legislation allowing such prescribing has existed for nearly 20 years.

As most of the studies included in this review were based in the USA and the UK, there is also a need for further research from other countries that have more recently adopted NMP models within their distinct healthcare systems.

### Strengths and limitations

The main strength of this study is its comprehensive nature as a review that maps the global evidence concerning the nature, impact, and factors influencing the implementation and delivery of NMP services for patients with mental illnesses. This study followed a systematic search strategy to identify relevant papers by using searching of multiple electronic databases and hand-searching of the reference lists of the included studies. The search process and the reporting of the findings for this scoping review followed established PRISMA-ScR guidelines with the Meta-Analyses Extension for Scoping Reviews [32]. To ensure the accuracy of the extraction process, a total of 20 out 63 of data was independently extracted and checked by two authors (RN. K, JH).

Despite this comprehensive search strategy, this review might be limited by not searching the grey literature, which might have introduced potential publication bias in terms of missing relevant papers; nevertheless, the priority was to identify high-quality evidence, which grey literature may not always provide [123]. Another limitation is that a quality assessment of the included studies was not conducted, as this scoping review aimed to identify and appraise a broad range of evidence. This review also restricted the included studies to those in English, which might have led to relevant studies conducted in non-anglophone countries being excluded, potentially narrowing the scope of this review. However, only one non-English study that was otherwise deemed potentially eligible for inclusion was excluded during the full-text screening stage [124].

## Conclusion

This scoping review was the first to identify and explore the published literature regarding the nature, impact, and factors influencing successful implementation and delivery of NMP for patients with mental illnesses. The review revealed some differences and similarities regarding the nature of NMP services across countries, including the medicines used, services delivered, and patient groups cared for. This review highlights the positive impact of NMPs in increasing service capacity, improving care access, enhancing multidisciplinary collaboration, and providing positive patient experiences. Nevertheless, evidence of impact remains limited in areas such as patient outcomes, medication management, and service delivery, and should be expanded for more comprehensive understanding. This review identified barriers to service implementation and delivery, such as inadequate training, supervision, and remuneration, which must be addressed to enable the full utilisation of NMP services. While valuable evidence on nurse-led non-medical prescribing for mental illness was provided, future studies should expand the evidence base for pharmacist NMPs and other healthcare professionals' NMPs, particularly in countries outside the UK and USA, which were under-represented. Addressing this gap is important given that their role is evolving in countries such as the UK.

## Supplementary Information

Additional file 1: Embase search strategy. The full electronic search strategy for the Embase database, including limits used.

Additional file 2: PRISMA-ScR Checklist. The checklist used for reporting items for systematic reviews and meta-analyses extension for scoping reviews.

Additional file 3: Data extraction form. This extraction form used during the extraction process include the key information to be extracted from each included study.

Additional file 4: Types of supervised practising. Description of different types of supervised practising.

## Data Availability

No datasets were generated or analysed during the current study.

## Abbreviations

AD: Alzheimer’s Disease
ADHD: Attention Deficit Hyperactivity Disorder
APPNs: Advanced Practice Psychiatric Nurses
ASD: Autism Spectrum Disorder
AUD: Alcohol Use Disorder
BPRS-E: Brief Psychiatric Rating Scale-Expanded Version
CAT: Crisis, Assessment, Treatment
CC: Community Care
CD: Conduct Disorder
CGI: Clinical Global Impression of Improvement Scale
CAYP: Child and Young People
CMHD: County Mental Health Director
CNS: Central Nervous System
CPD: Clinical Professional Development
CPN: Community Psychiatric Nurses
CSQ-8: Client Satisfaction Questionnaire (Eight-Item)
EDS: Edinburgh Depression Scale
ED: Emotional Disorder
ETOH: Ethyl Alcohol
FBM: Financial and Billing Manager
GAD-7: Generalised Anxiety Disorder
GPs: General Practitioners
HC: Hospital Care
HRM: Human Resources Manager
ICU: Intensive Care Unit
IP: Independent Prescribing
LA: Long Acting
MARS: Medication Adherence Report Scale
MDD: Major Depression Disorder
MG: Medical Group
MHTs: Mental Health Teams
MHMD: Mental Health Medical Director
MDT: Multidisciplinary Team
NA: Not Available
NG: Nurse Group
NMP: Non-Medical Prescribing
NMPs: Non-Medical Prescribers
NP: Nurse Prescriber
NS: Not Stated
PCPs: Primary Care Providers
PHQ-9: Patient Health Questionnaire
PMHNPs: Psychiatric Mental Health Prescribers
PP: Pharmacist Prescriber
PGD: Patient Group Direction
PSs: Psychiatric Specialists
PSTD: Post-Stress Traumatic Disorder
PRISMA-ScR: Preferred Reporting Items for Systematic Reviews and Meta-Analyses Extension for Scoping Reviews
QM: Quality Manager
RACFs: Residential Aged Care Facilities
SA: Short Acting
SC: Senior Clinicians
SDQ: Strengths and Difficulties Questionnaire
SE: Side Effect
SIMS: Satisfaction with Information about Medicines Scale
SP: Supervised Prescribing
SPC: Specialist Palliative Care
SNRIs: Serotonin-Norepinephrine Reuptake Inhibitors
SSRI: Selective Serotonin Reuptake Inhibitors
SUD: Substance Use Disorder
TCA: Tricyclic Antidepressant
WSAS: Work and Social Adjustment Scale
