# Supplementary material for: Exploring non-medical prescribing for patients with mental illness: a scoping review
Source: BMC Psychiatry. 2025 May 19;25:504. doi: 10.1186/s12888-025-06938-6 (PMC12090459; doi:10.1186/s12888-025-06938-6)
Supplement: Supplementary file 1 — Additional file 1: Embase search strategy. The full electronic search strategy for the Embase database, including limits used. [file 12888_2025_6938_MOESM1_ESM.docx]

Embase <1980 to 2024 Week 27>

1 Prescrib*.mp.

2 Nurs*.mp.

3 pharmacist*.mp.

4 practitioner*.mp.

5 paramedic.mp.

6 non medical.mp.

7 independent.mp.

8 supplementary.mp.

9 2 or 3 or 4 or 5 or 6 or 7 or 8

10 Mental health*.mp.

11 Psychiatr*.mp.

12 Mental illness*.mp.

13 Schizophrenia.mp.

14 Dementia.mp.

15 Bipolar disorder*.mp.

16 Psychosis.mp.

17 Depressi*.mp.

18 Anxiety disorder*.mp.

19 Mood disorder*.mp.

20 Attention deficit hyperactivity disorder*.mp.

21 Eating disorder.mp.

22 Insomnia.mp

23 10 or 11 or 12 or 13 or 14 or 15 or 16 or 17 or 18 or 19 or 20 or 21 or 22

24 develop*.mp.

25 Assess*.mp.

26 Examin*.mp.

27 Describ*.mp.

28 Evaluat*.mp.

29 Explor*.mp.

30 Understand*.mp.

31 Investigat*.mp.

32 Identif*.mp.

33 look*.mp.

34 review*.mp.

35 impact.mp.

36 Analy*.mp.

37 explain*.mp.

38 discuss*.mp.

39 outlin*.mp

40 Elicit*.mp.

41 24 or 25 or 26 or 27 or 28 or 29 or 30 or 31 or 32 or 33 or 34 or 35 or 36 or 37 or 38 or 39 or 40

42 1 and 9 and 23 and 41

43 limit 42 to yr="2003 -Current"
