# Supplementary material for: Exploring non-medical prescribing for patients with mental illness: a scoping review
Source: BMC Psychiatry. 2025 May 19;25:504. doi: 10.1186/s12888-025-06938-6 (PMC12090459; doi:10.1186/s12888-025-06938-6)
Supplement: Supplementary file 3 — Additional file 3: Data extraction form. This extraction form used during the extraction process include the key information to be extracted from each included study. [file 12888_2025_6938_MOESM3_ESM.docx]

| Title | Author/s | Study country | Publication year | Data collection | Duration of the study | Study setting | Study design (qualitative, quantitative) | Aim/objectives of the study | Study population (profession of NMPs) | Study population (number of NMPs |
| --- | --- | --- | --- | --- | --- | --- | --- | --- | --- | --- |
|  |  |  |  |  |  |  |  |  |  |  |

| Role of NMPs (what medication) | Role of NMPs (what service) | Role of NMPs (what mental illness patient had) | Role of NMPs (model of prescribing) | The impact of NMPs | Facilitators to NMPs | Additional information |
| --- | --- | --- | --- | --- | --- | --- |
|  |  |  |  |  |  |  |
