# Supplementary material for: Exploring non-medical prescribing for patients with mental illness: a scoping review
Source: BMC Psychiatry. 2025 May 19;25:504. doi: 10.1186/s12888-025-06938-6 (PMC12090459; doi:10.1186/s12888-025-06938-6)
Supplement: Supplementary file 4 — Additional file 4: Types of supervised practising. Description of different types of supervised practising. [file 12888_2025_6938_MOESM4_ESM.docx]

| Type of Model | Description |
| --- | --- |
| Supplementary Prescribing | A form of prescribing where non-medical prescribers work in partnership with an independent prescriber (usually a doctor or dentist) to implement an individualized clinical management plan for a patient, with the patient's agreement. |
| Restricted Practice | A model in which a nurse or pharmacist must work under the direct supervision of a physician, with limited autonomy in their practice. |
| Reduced Practice | A practice model with fewer restrictions, where a nurse or pharmacist operates with moderate autonomy without requiring constant supervision from a physician. |
| Collaborative Practice Agreement | A scope of practice based on a formal agreement between an independent prescriber and a nurse or pharmacist, outlining their specific roles and responsibilities within the collaboration. |

Supplementary file 3
